# Correction: Providing open-label placebos remotely—A randomized controlled trial in allergic rhinitis

**DOI:** 10.1371/journal.pone.0252850

**Published:** 2021-06-02

**Authors:** Tobias Kube, Verena E. Hofmann, Julia A. Glombiewski, Irving Kirsch

Following the publication of this article [1], concerns were raised regarding the record number provided for the ISRCTN registration of this study. The record number 39018 reported in the article refers to an incomplete ISRCTN registration application, as opposed to a published ISRCTN entry. The retrospective registration of this trial has now been completed, and this correction notice is issued to update the ISRCTN registration details to trial ID: ISRCTN11274834.

## References

[1] Kube T, Hofmann VE, Glombiewski JA, Kirsch I (2021) Providing open-label placebos remotely—A randomized controlled trial in allergic rhinitis. PLoS ONE 16(3): e0248367. 10.1371/journal.pone.0248367 33705475 PMC7951912

