# Supplementary material for: Providing open-label placebos remotely—A randomized controlled trial in allergic rhinitis
Source: PLoS One. 2021 Mar 11;16(3):e0248367. doi: 10.1371/journal.pone.0248367 (PMC7951912; doi:10.1371/journal.pone.0248367)
Supplement: S1 Table — (DOCX) [file pone.0248367.s002.docx]

S1 Table.

*Items of the extended Health Screening Experience Questionnaire*

| **Item** | **Subscale** |
| --- | --- |
| 1. The provider appeared confident in the conversation. | Competence |
| 1. The provider gave me a good feeling. | Warmth |
| 1. The provider handled the medical aspects in the conversation well. | Competence |
| 1. The provider appeared confident when asking about and explaining the medical aspects. | Competence |
| 1. The provider responded well to me. | Warmth |
| 1. The provider was friendly when talking to me. | Warmth |
| 1. The provider was competent. | Competence |
| 1. The provider was warm and cordial. | Warmth |
| 1. The provider was professional. | Competence |
| 1. The provider listened attentively. | Warmth |
| 1. The provider made sure that I felt well. | Warmth |
| 1. The provider appeared friendly to me. | Warmth |
| 1. The provider provided information understandably. | Competence |
| 1. The provider answered my questions well. | Warmth |
| 1. It seemed important to the provider to respond to me. | Warmth |
| 1. The provider did not seem interested in me as a person, but paid more attention to the procedure.* | Warmth |
| 1. The provider seemed a little hectic.* | Warmth |
| 1. The provider left room for questions. | Warmth |
| 1. The provider was affectionate and empathic. | Warmth |
| 1. The provider held much eye-contact. | Warmth |
| 1. The provider expressed understanding for my situation. | Warmth |

*Note.* Items marked with * are reversely scored.

**Open-ended questions to assess knowledge about placebos**

1. Do placebos contain active ingredients?
2. Are placebos the same as common pharmaceuticals?
3. For what conditions have placebos been shown to be effective?
4. How often should placebos be taken?
5. What might be reasons for why placebos have measurable effects?
6. Is a positive attitude towards placebos necessary for them to work?

**Treatment expectancies***

1. I am confident that the placebos will help me.
2. The placebos will help me to cope with my symptoms.
3. I think that the placebos will make me less sensitive in the pollen season.
4. The placebos will alleviate my allergic symptoms.
5. I expect that I belong to the people for whom placebos are effective.

* rated on a 5-point Likert scale from 1 = “I don´t agree at all” to 5 = “I completely agree”

**COVID-19 items***

1. I am concerned that my symptoms could be related to COVID-19 reported hay fever symptoms are connected to a Covid-19 infection.
2. I am confident that my symptoms are independent from a COVID-19.

* rated on a 4-point Likert scale from 1 = “I don´t agree at all” to 4 = “I completely agree”
