# Supplementary material for: Providing open-label placebos remotely—A randomized controlled trial in allergic rhinitis
Source: PLoS One. 2021 Mar 11;16(3):e0248367. doi: 10.1371/journal.pone.0248367 (PMC7951912; doi:10.1371/journal.pone.0248367)
Supplement: S1 File — (PDF) [file pone.0248367.s003.pdf]

Institutional Review Board of Department 8 Psychology, University of Koblenz-Landau

Standard questionnaire for the evaluation of a research project

(based on the ethical guidelines of the DGP)

**A. Title/Name of the research project**
How do open label placebos work? A randomized clinical trial on the role of clinical interaction style in hay fever

**B. Name and address of the principal investigator (supervisor)**	
Last name, first name:	Kube, Tobias
Address:	Ostbahnstr. 10, 76829 Landau
Phone number:	+49 6341-28035652
E-Mail address:	kube@uni-landau.de

C. Brief project description (max. 500 words)
(goals, sample of participants, method, scientific knowledge gain)

Background

The opinion held for a long time that deception of patients is necessary for placebos to have a positive effect is based on current knowledge no longer sustainable [1-3]. Several studies have already demonstrated the positive effect of open label placebos (OLP) on healthy study participants [2] as well as clinical samples of patients with irritable bowel syndrome [3] or chronic pain [4]. A positive effect of OLP on patients struggling with hay fever was also already observed [5]. The psychological mechanisms to explain placebo effects of this sort are however still mostly unknow or lack explanatory power [6]. Multiple past studies were able to demonstrate that detailed information or a rational about the effects of placebos can support their effectiveness [2,7]. However, it must be assumed that additional mechanisms exist that can enhance or weaken the effectiveness of placebos. Therefore, this proposed research endeavor aims to examine the influence of the interaction between patients and practitioners. Early evidence that indicates a higher effectiveness of placebos using a warm, empathetic interaction style in OLP settings compared to a limited style already exists [8,9].

Goals

The aim of the proposed research project is to investigate whether a practitioner´s clinical interaction style influences the effect of open label placebos on patients with hay fever syndrome. For this purpose, the administration of placebos as well as the clinical interaction style is experimentally varied.

Method

A total of 80 people that suffer from hay fever syndrome / allergic rhinitis are to be examined for the proposed study. People who are already taking other medication are included into the sample, though medication intake is not a requirement. The medication should not to be altered during the placebo intake.
The design of a study by Schaefer and colleges [1] functions a fundamental paradigm and gets adapted for the proposed project. Patients who are according to self-report struggling with hay fever get invited to an initial interview, where their symptoms are examined and a placebo rational is imparted. Prior to their arrival participants are randomly assigned to an interaction style. After the interview participants are randomly assigned to one of the placebo groups. Study participants of the active placebo group are to take one placebo pill twice a day during the following two weeks. At the end of the two weeks another examination of the hay fever symptoms of all participants takes place.

Scientific knowledge gain

Allergies and hay fever are widespread globally and impair the quality of life of those affected [4, 5]. Placebos present a promising treatment option [12]. The underlying mechanisms of the placebo effect are still mostly unknown though [6]. This proposed research project is hoped to not only enhance the

knowledge about the effects of OLP but also have implications on the treatment of allergies and provide an explanation for another possible mechanism to enhance placebo effectiveness.

**D. Are further researchers involved in the project?**	
Names/Faculty:	B.Sc. Verena Hofmann, University of Koblenz-Landau, Department of Psychology, Working group Clinical Psychology and Psychotherapy<br>Prof. Dr. Julia Anna Glombiewski, University of Koblenz-Landau, Department of Psychology, Working group Clinical Psychology and Psychotherapy<br>Prof. Dr. Ted Kaptchuk, Harvard Medical School, Program in Placebo Studies, Boston, USA<br>Prof. Dr. Irving Kirsch, Harvard Medical School, Program in Placebo Studies, Boston, USA

**E. Funding?**		Yes	No
Source of funding:			**x**
Names**:**			
Self-funded (as part of a thesis):		**x**	

**F. Does the source of funding request an ethical statement?**	Yes	No

**G. Enclosed with this application (as a pdf file please):** ***Please check all that apply***	
Declaration of informed consent (mandatory)	x
Information and education for study participants (mandatory)	x
Declaration whether this application has previously been submitted to another institutional review board. If so, enclose a statement	x
Declaration of no objection of another institutional review board	
All steps of the research process listed in tabular form including a compilation of the type and number of study participants, their recruitment, instructions, tasks, questionnaires (names only), instruments used in the study	x
Declaration of consent for video and/or sound recording	
Debriefing concerning studies with active or passive deception	

Note:
Details on the declaration of consent and information for study participants can be found in the ethical guidelines of the DGP https://www.dgps.de/fileadmin/documents/Empfehlungen/berufsethische_richtlinien_dgps.pdf (3a-e; 6;9) as well as the DGP guidelines for submitting applications (point C): https://www.dgps.de/index.php?id=186

**H. Research Study Checklist**		
	Yes	No
**1. Voluntariness:**<br>Is the voluntariness of participation guaranteed?	x	
**2. Capacity to contract:**<br>Will people take part in the study who cannot give their consent to participate because they are underage, have limited capacity or incapacity of judgement (e.g. babies, toddlers, people under the age of 18, people who are legally incapable of giving consent)?		x
**3. Impaired groups of people:**<br>Will people take part in the study who belong to a particularly vulnerable group (e.g. clinical samples, people with learning difficulties, people in clinic or prison settings, people with dementia, people in retirement homes, people with physical disabilities)?	x	

**4. Termination:**<br>Are the study participants assured that they can terminate the examination at any time without giving reasons and without negative consequences?	x	
**5. Inclusion and exclusion criteria:**<br>Are there inclusion and exclusion criteria for the study participants?	x	
**6. Informed consent:**<br>Is informed consent declared in written form?	x	
**7. Information:**<br>Are the study participants fully informed about the aims and purposes of the study?	x	
**8. Deception concerning participation:**<br>Is it necessary that people take part in the study without being informed about their participation at that time or without having given their consent (e.g. experimental field investigations, concealed observation) or that they are not sufficiently informed about the purpose and content of the study (does not include disclosure of hypotheses)?		x
**9. Active deception concerning content, purpose, method or setting:**<br>Are people actively and purposefully deceived about the content, purpose, method and / or setting of the study (e.g. by leading them to believe in a fake purpose, giving wrong information, withholding important information etc.)?		x
**10. Intimacy / Stigmatization:**<br>Are questions asked that are of an intimate nature for the respondents (e.g. debilitating personal experiences, sexuality) or the answers to which can be perceived as stigmatizing (e.g. illegal or deviant behavior such as drug use, addictions, the abuse of luxury food or even political beliefs)?		x
**11. Psychological stress:**<br>Is it to be expected that the participants will experience psychological stress, fear, exhaustion or other negative effects as a result of the study?		x
**12. Physical risks:**<br>Are the study participants faced with any invasive measurements? Do they undergo any potentially debilitating (e.g. blood or saliva sampling) or potentially harmful procedures? Is physical pain caused? Are side effects to be expected?		x
**13. Substance administration:**<br>Are any medications, placebos or other substances administered to the study participants?	x	
**14. Confidentiality:**<br>Are personal data treated confidentially and saved anonymously?	x	
**15. Data protection:**<br>Is the data security of personal data guaranteed?	x	
**16. Information on data protection:**<br>Are the participants informed about the data security of their personal data?	x	
**17. Right to data deletion:**<br>Can the participants at any time request the deletion / obliteration of their personal data from an existing code list and are they informed about this right? If there is no code list, can the deletion be requested immediately after the examination?	x	
**18. Insurance coverage:**<br>Do the participants have a travel insurance or are they informed that their journey is not covered?	x	

If you have marked one or more of the highlighted fields in the basic questionnaire checklist with a cross, please submit an extensive application with all test materials. Within the extensive application please especially focus on the questions that you ticked the highlighted field for and explain why these aspects of the study are required and how the ethical guidelines will be abided in these areas. Additionally please address the cost-benefit aspect of the study.

Note:
Further information concerning particular topics can be found on the following website: http://www.dgps.de/index.php?id=185

I confirm that all information in this application is correct and in accordance with the guidelines of the DGP´s institutional review board. I also confirm that the information provided does not differ from the information in the application to the funding institution concerned. I understand that the ultimate responsibility for compliance with the guidelines rests with me.

Landau, 17.12.2019

Place, Date

Signature of the principal investigator / supervisor

Annex:

- Appendix A: Study information
- Appendix B: Declaration of informed consent
- Appendix C: Study design
- Appendix D: Questionnaires used
- Appendix E: Final information
- Appendix F: Reasoning behind ethically potentially problematic decisions
- Appendix G: Safety votes on previous research projects
- References

UNIVERSITÄT KOBLENZ · LANDAU

A study on the effect of placebos on hay fever

Study information

Principal investigator:

Dr. Tobias Kube
University of Koblenz-Landau
Working group Clinical Psychology and Psychotherapy
Ostbahnstr. 10, 76829 Landau
E-mail: kube@uni-landau.de

Dear prospective study participants,

Our working group is currently conducting a study on the effects of placebos on hay fever. We would like to share some information about this study with you below and kindly ask you to participate in this project.

What is the study about?
"Placebo effects" are positive effects of treatments that do not contain any medically or pharmacologically active ingredients. For a long time, it was assumed that placebo effects can only occur when patients are (incorrectly) made believe that they are administered a "real" drug. Recent research has challenged this assumption: It is now known that open label placebos can also have positive effects. In our study we want to investigate the effects of open label placebos on allergic rhinitis (commonly known as "hay fever") and understand how their effects come about.

What does participating in the study mean for me?
When you participate in the study you are randomly assigned to either the placebo group or the control group. If you were assigned to the placebo group, we would ask you to take placebo pills twice a day for two weeks. At the end of the two weeks there would be another interview here in Ostbahnstr., where you would be asked about your experience taking the placebo and to fill out a questionnaire concerning the development of your symptoms. If you were assigned to the control group, you would not receive any treatment for the first two weeks. After two weeks there would be another appointment, as with the placebo group, where you would be asked about the development of your symptoms. If you wish you could receive the placebo treatment twice a day for two weeks after this interview as well. At the end of these two weeks another appointment would take place where you would be asked about your experience with the placebo. Each appointment with us will take around 30 minutes. In case we observe clinically relevant symptoms in the course of our survey, we can inform you about them if you wish. If you would like this kind of feedback, please talk to the principal investigator.
`

Why should I take part in the study?
By participating in the study, you support the quest of getting a better understanding of how open label placebos work. Based on previous studies on open label placebos in hay fever treatment there is a realistic chance that placebo treatment can have positive effects on the development of your symptoms. By participating, you will also contribute to increase the understanding of a reformed hay fever treatment.

Possible disadvantages and risks
A big advantage of placebos is that they do not cause any side effects. Besides the required time no disadvantages are to be expected from participating in this study.

Data protection and voluntariness

Participation in the study is voluntary. Withdrawal from your consent to participate is possible at any time without giving reasons and is not associated with any disadvantages. The collected data is pseudonymized and treated strictly confidentially in accordance with the rules of data protection (Federal Data Protection Act §40) and confidentiality. This means you are assigned a code on a list where your data is saved electronically. This code list is kept under lock and key. As soon as the data collection has been completed (by 30.06.2021 at the latest) the allocation key will be destroyed and your data will exist in anonymized form. This means it will no longer be possible to draw conclusions form the data to you as a person. The anonymized data will be stored for 10 years in accordance with legal provisions. As long as the allocation key exists, i.e. until the data collection has been completed, you can request the deletion (or restriction of processing) of your collected data at any time. After that your name can no longer be assigned to your data, so deletion is no longer possible. Your data will only be used for evaluations as part of this study. The anonymized results of the study can be part of scientific publications. Your declaration of consent will be kept separate from the collected data and will be destroyed after the study has been completed (by 30.06.2020 at the latest).

If you have any further questions concerning our study please do not hesitate to contact us. This is the contact information of the principal investigator:

Dr. Tobias Kube

University of Koblenz-Landau

Working group Clinical Psychology and Psychotherapy
Ostbahnstr. 10, 76829 Landau
E-mail: kube@uni-landau.de
Phone number: 06341-28035652

A study on the effect of placebos on hay fever

Declaration of informed consent

Principal investigator:

Dr. Tobias Kube
University of Koblenz-Landau
Working group Clinical Psychology and Psychotherapy
Ostbahnstr. 10, 76829 Landau
E-mail: kube@uni-landau.de

I have been informed about the nature, meaning and consequences of the study led by Dr. Tobias Kube. I have had ample opportunity to gather information about the study from the test instructor and ask and any questions I had. They were answered in an understandable way by the test instructor. I am aware that I can contact the principal investigator with further questions at any time using the contact information above.

I am aware that this study is primarily intended to expand scientific knowledge and may not be of any personal benefit to me. I know that I can contact the principal investigator in case I want to be informed about potential clinically relevant findings.

With my signature I declare that I have understood the procedure and information and participate voluntarily in this study. I understand that I can withdraw from my participation at any time without giving any reason and without inflicting any personal disadvantages on myself. The test instructor can also end the study at any time.

I am aware that my data will be pseudonymized and will be treated with strict confidentiality in accordance with the rules of data protection (Federal Data Protection Act §40) and confidentiality. This means that I will be assigned a code on a list which is how my data will be saved electronically. This assignment list will be kept under lock and key. As soon as the data collection has been completed (by 30.06.2021 at the latest) the allocation key will be destroyed and my data will exist in anonymized form. This means that it will no longer be possible to draw conclusions from the data to my person. The anonymized data will be saved for 10 years in accordance with legal provisions. As long as the allocation key exists, i.e. until the data collection has been completed, I can request the deletion (or restriction of processing) of my individual data at any time. After that, my name can no longer be assigned to the data so a deletion will no longer be possible. My data will only be used for evaluations as part of the study. The randomized results of the study can be part of scientific publications.

I have the right

- to receive information (in accordance with Art. 15 DS-GVO), including the provision of a copy free of charge,
- to request a correction (in accordance with Art. 16 DS-GVO) if necessary,

- to request a deletion if necessary, as long as there are no retention obligations (in accordance with Art. 17 DS-GVO),
- to request the restriction of processing (in accordance with Art. 18 DS-GVO),
- to transmit the data to another person without hindrance (data transmission in accordance with Art. 20 DS-GVO),
- to object to the use for scientific purposes (beyond the direct purposes of the study).

Contact information of the principal investigator:
Dr. Tobias Kube, University of Koblenz-Landau, working group Clinical Psychology and Psychotherapy, Ostbahnstr. 10, 76829 Landau, e-mail: kube@uni-landau.de, phone number: 06421-2823341

Contact information of the data protection supervisory authority of Rhineland-Palatinate:
Der Landesbeauftragte für den Datenschutz Rheinland-Pfalz, Hintere Bleiche 34, 55116 Mainz
Mailing address:
Postfach 30 40
55020 Mainz

Contact information of the data protection officer of the Landau Campus:
Universität Koblenz-Landau, Datenschutzbeauftragte Dr. Susanne Weis, Campus Landau, Fortstr. 7, 76829 Landau, datenschutz@uni-landau.de

I hereby declare that I was informed about the aims and processes of the study and have understood them. I hereby voluntarily declare my participation in the study.

Last name, first name:____

______________________________	______________________________
Place, date and signature of the principal investigator	Place, date and signature of the study participant

Appendix C: Study design and procedure

Design

It is a 2x2 design, in which treatment (placebo vs. no treatment) as well as clinical interaction style (enriched vs. limited) are varied.

Pretest

At the beginning the participants´ symptoms and the symptom severities are examined by using the questionnaire developed by Schaefer and colleges [7]. Afterwards the participants are given a placebo rational and information about the following procedure. Understanding, especially of the characteristics of placebo pills, is queried afterwards to ensure that the participants know that they will not be administered any medically or pharmacologically active substances. This is followed by a random allocation to one of the two treatment conditions: Placebo vs. No Treatment (NT) control group and if applicable the dispense of the placebo pills for the upcoming two weeks. The following information on placebos and placebo effects will be provided for participants assigned to the placebo group:

- In two previous studies (Schäfer et al., 2016, 2018) an open label placebo has proven to be an effective treatment option for hay fever, as it influenced the symptoms in a positive way.
- Various aspects are being discussed as contributors to this effect, including learning mechanisms such as classical conditioning.
- While it can help to have positive expectations towards the use of placebos, it is also perfectly normal to be skeptical about placebos.
- In terms of adherence, participants are told that it is very important that they take the placebo twice a day during the study period, as previous research has shown that placebos have a beneficial effect on symptom progression, especially when taken regularly.
- Finally, the study participants are told that people´s reactions to placebos can vary in fortitude and pace, and it is therefore possible that they will not notice an effect right after taking the first dose.

Experimental conditions

Clinical interaction style is varied based on the characteristics listed below. It is important for the applicant to point out that the limited condition is not designed as a conscious negative example with regard to the clinical interaction. This means the goal is not to artificially attempt to construct a particularly unfavorable clinical encounter. Rather, it is planned that the limited condition represents a neutral, factual conversation, while positive features are added in the enriched condition and the clinical interaction is designed especially warm and personal.

Factor	Limited	Enriched
**Verbal Communication**	• Brief greeting without introduction by name<br>• Little feedback / queries<br>• Ask few questions<br>• Conversation has a more standardized interview character with focus on procedure<br>• Sober, factual language	• Personal greeting with introduction by name<br>• Space for queries and feedback; Ask questions<br>• Avoid or explain technical terms<br>• Patient-oriented, empathetic and friendly language<br>• Questions concerning symptoms and quality of life<br>• Praise previous attempts to reduce symptoms<br>• Gratitude for participation
**Non-verbal Communication**	• Little eye contact, fill in form on the side<br>• Few gestures, few facial expressions (neutral facial expression)<br>• Obligation and factual oriented impression; General atmosphere rather sober	• A lot of eye contact<br>• upright, facing posture<br>• affirming, validating gestures and facial expressions, friendly smile<br>• Friendly greeting with handshake<br>• Person-oriented setting; General atmosphere warm and comforting
**Surrounding or contextual factors**	• Sitting position behind desk, patient sitting in front of it<br>• No name tag<br>• Impersonal gown<br>• No personal items in the room, sterile treatment room	• Sitting position at a shared meeting table<br>• Name tag<br>• Glass of water on the table, possibly with a carafe<br>• Flowers on the table<br>• In the background bookshelf with specialist literature<br>• Private pictures in the background on bookshelf<br>• Gown, but personal clothing style recognizable

Corresponding with the variation in clinical interaction there are two NT control groups (each n = 20). Both control groups do not take placebos. The first NT control group receives an enriched initial interview to assess symptoms, symptom severity and impairment by symptoms in daily life and a final interview two weeks later. The second NT control group will have a limited initial interview with the same content and a final interview two weeks later as well.

This is contrasted by the two groups receiving a placebo (each n = 20). Both active experimental groups take a placebo pill twice a day for two weeks after the initial interview. The first experimental group receives an enriched initial interview as well as a final interview at the end of the placebo intake with the same content as the interviews of the control groups. The second experimental group will have a limited initial interview and a final interview at the end of the placebo intake with the same content as with interviews of the control groups.

<u>Posttest</u>

After two weeks during with the participants of the active placebo condition took a placebo pill twice a day and the participants of the NT control group did not receive any placebo treatment, symptoms are assessed again using the questionnaire by Schaefer and colleges [7]. Additionally, a manipulation check is performed where the participants evaluate their interaction with the practitioner in a questionnaire. Leftover placebo pills are handed back to the practitioner.

Appendix D: Questionnaires used

- Questions on hay fever symptoms [5, 7]
- Adapted version of the Pain Disability Index (PDI) to assess the impairment caused by hay fever symptoms in everyday life
- PHQ-9 as a screening instrument for depressive symptoms
- Questions concerning the perception of the clinical interaction style
- Questions on the treatment expectations
- Questions on knowledge about placebos
- Sociodemographic questions about age, gender, level of education, occupation, marital status

A study on the effect of placebos on hay fever

Final information

Principal investigator:
Dr. Tobias Kube
University of Koblenz-Landau
Working group Clinical Psychology and Psychotherapy
Ostbahnstr. 10, 76829 Landau
E-mail: kube@uni-landau.de

Dear study participants,

thank you for having taken part in our study. We would like to give you some final information about our study.

In our study we are striving to examine how the effect of open label placebos is influenced by the interaction between patient and practitioner. We therefore have tested two types of clinical interaction within this study: a factual, less personalized interaction and an enriched, particularly warm and personalized interaction. It was decided at random whether you received one or the other interaction style in conversation with your practitioner.

If you have any further questions concerning the study, please do not hesitate to contact us.

Appendix F: Reasoning behind ethically potentially problematic decisions

Impaired sample as well as inclusion and exclusion criteria

Only people who are according to self-report affected by hay fever symptoms should take part in this study. Previous studies [1] have demonstrated that OLP can be effective in this sample. The participation of impaired people can help to derive interventions that can optimize the treatment options for this group in the future. This is especially important considering the lack of preventative measures available and the simultaneous increase in prevalence rates in allergic diseases [10].

The above explains the need for inclusion criteria to investigate this sample.

The inclusion criteria are:

- Self-reported hay fever symptoms
- Minimum age of 18
- Sufficient German language skills

Sufficient German language skills are necessary since the majority of the experimental manipulation is language based, so sufficient language knowledge is crucial.

Substance administration

An essential part of this study is the administration of placebo pills to half of the study participants, while the other half receives none. This is necessary to investigate the main effects of substance and clinical interaction as well as the interaction of both. It is explained to the participants in advance that placebos are pharmacologically ineffective tablets, so they can give their consent fully informed. The composition of the placebo pills does not pose any health risks.

Appendix G: Safety votes on previous research projects

Over the last few years our working groups has conducted several studies on placebo and nocebo effects at the Phillips University of Marburg. These were assessed as ethically harmless by the institutional review board concerned. The two most recent studies investigating the effect of open label placebos were as followed:

- Project names: "Hope and expectations as effect factors in open label placebos – An experimental study on pain perception"; "Effect factors in open and closed label placebos – An experimental study on the induction of sadness"
- Institutional review boards concerned: Institutional review board of the Department of Psychology at the Phillips University of Marburg

References

1. Kaptchuk, T. J. (2018). Open-label Placebo: Reflections on a research agenda. Perspectives in Biology and Medicine, 61, 311-334. doi: 10.1353/pbm.2018.0045

2. Locher, C., Nascimento, A. F., Kirsch, I., Kossowsky, J., Meyer, A., & Gaab, J. (2017). Is the rationale more important than deception? A randomized controlled trial of open-label placebo analgesia. PAIN, 0, 1-9. doi: 10.1097/j.pain. 0000000000001012

3. Kaptchuk, T. J., Friedlander, E., Kelley, J. M., Sanchez, M. N., Kokkotou, E., Singer, J. P., … & Lembo, A. J. (2010). Placebos without deception: A randomized controlled trial in irritable bowel syndrome. PLoS ONE, 5, 1-7. doi: 10.1371/journal.pone.0015591

4. Carvalho, C., Caetano, J. M., Cunha, L., Rebouta, P., Kaptchuk, T. J., & Kirsch, I. (2016). Open-label-placebo treatment in chronic low back pain: A randomized controlled trial. Pain, 157, 2766-2772. doi: 10.1097/j.pain. 0000000000000700

5. Schaefer, M., Harke, R., & Denke, C. (2016). Open-label placebos improve symptoms in allergic rhinitis: A randomized controlled trial. Psychotherapy and Psychosomatics, 85, 373-374. doi: 10.1159/000447242

6. Kaptchuk, T. J., & Miller, F. G. (2018). Open label placebo: Can honestly prescribed placebos evoke meaningful therapeutic benefits? British Medical Journal, 363, 1-3. doi: 10.1136/bmj.k3889

7. Schaefer, M., Sahin, T., & Berstecher, B. (2018). Why do open-label placebos work? A randomized controlled trial of an open-label placebo induction with and without extended information about the placebo effect in allergic rhinitis. PLoS ONE, 13(3), 1-14. doi: 10.1371/journal.pone.0192758

8. Enck, P., Bingel, U., Schedlowski, M., & Rief, W. (2013). The placebo response in medicine: Minimize, maximize or personalize? Nature Reviews Drug Discovery, 12, 191-204. doi: 10.1038/nrd3923

9. Kaptchuk, T. J., Kelley, J. M., Conboy, L. A., Davis, R. B., Kerr, C. E., Jacobson, E. E., … & Lembo, A. J. (2008). Components of placebo effect: Randomised controlled trial in patients with irritable bowel syndrome. British Medical Journal, 336, 999-1003. doi: 10.1136/bmj.39524.439618.25

10. Bergmann, K.-C., Heinrich, J., & Niemann, H. (2015). Current status of allergy prevalence in Germany. Allergo Journal International, 25, 6-10. doi: 10.1007/s40629-016-0089-1

11. Seidman, M.D., Gurgel, R.K., Lin, S.Y., Schwartz, S.R, Baroody, F.M., Bonner, J.R., … & Nnacheta, L.C. (2015). Clinical practice guideline: Allergic rhinitis. Otolaryngology – Head and Neck Surgery, 152, S1-S43. doi: 10.1177/0194599814561600

12. Wedi, B. (2019). Der Placeboeffekt in der Allergologie: Die ideale Therapieoption? Allergolgoie, 42, 155-156. doi: 10.5414/ALX02084e

13. Enck, P., Bingel, U., Schedlowski, M., & Rief, W. (2013). The placebo response in medicine: Minimize, maximize or personalize? Nature Reviews Drug Discovery, 12, 191-204. doi: 10.1038/nrd3923

14. Hoenemeyer, T. W., Kaptchuk, T. J., Mehta, T. S., & Fontaine, K. R. (2018). Open-label placebo treatment for cancer-related fatigue: A randomized-controlled clinical trial. Scientific Reports, 8, 2784-2791. doi: 1038/s41598-018-20993-y
