# Supplementary material for: Providing open-label placebos remotely—A randomized controlled trial in allergic rhinitis
Source: PLoS One. 2021 Mar 11;16(3):e0248367. doi: 10.1371/journal.pone.0248367 (PMC7951912; doi:10.1371/journal.pone.0248367)
Supplement: S2 File — (PDF) [file pone.0248367.s004.pdf]

Lokale Ethikkommission des FB 8 Psychologie, Universität Koblenz-Landau

Basisfragebogen zur Beurteilung eines Forschungsvorhabens
(unter Zugrundelegung der Ethik-Richtlinien der DGPs)

**A. Titel/Bezeichnung des Forschungsvorhabens**
Wie wirken offen verabreichten Placebos? Eine randomisiert-kontrollierte Studie zur Rolle des klinischen Interaktionsstils bei Heuschnupfen

**B. Name und Anschrift des hauptverantwortlichen Wissenschaftlers (Betreuer)**	
Name, Vorname:	Kube, Tobias
Anschrift:	Ostbahnstr. 10, 76829 Landau
Telefon-Nr.:	+49 6341-28035652
E-Mail:	kube@uni-landau.de

C. Kurze Zusammenfassung des Forschungsvorhabens (max. 500 Wörter)
(Ziele, Pbn-Stichprobe, methodisches Vorgehen, wiss. Erkenntnisgewinn)

Hintergrund

Die lange Zeit verbreitete Meinung, dass für eine positive Wirkung von Placebos eine Täuschung der Patient*innen nötig sei, ist nach dem heutigen Kenntnisstand nicht länger vertretbar [1-3]. So konnte bereits in mehreren Studien an verschiedenen Stichproben, darunter sowohl gesunde Probanden [2] als auch klinische Stichproben wie Patient*innen mit Reizdarmsyndrom [3] oder chronischen Schmerzen [4], ein positiver Effekt offen verabreichter Placebos (OLP) nachgewiesen werden. Auch bei einer Stichprobe von Heuschnupfen-Patient*innen wurde bereits ein positiver Effekt von OLP gefunden [5]. Allerdings sind die psychologischen Mechanismen, die derartige Placeboeffekte erklären könnten, noch weitgehend ungeklärt oder besitzen keine ausreichende Erklärungskraft [6]. Bereits in mehreren Studien zeigte sich, dass das Vermitteln detaillierter Informationen bzw. eines Rationals über die Wirkung von Placebos deren Effekte unterstützen können [2,7]. Es ist allerdings davon auszugehen, dass es weitere Mechanismen gibt, die Placeboeffekte verstärken oder auch schwächen können. Daher soll in der geplanten Studie der Einfluss der Patient*innen-Behandler*innen-Interaktion überprüft werden. Erste Hinweise dafür, dass ein warmer, empathisch angereicherter Interaktionsstil im Vergleich zu einem limitierten Stil höhere Placeboeffekte in OLP-Settings erzielt, liegen bereits vor [8,9].

Ziele

In der geplanten Studie soll untersucht werden, inwiefern sich der klinische Interaktionsstil der Behandelnden auf den Effekt offen verabreichter Placebos bei Patient*innen mit Heuschnupfen Symptomen auswirkt. Dazu wird neben der Verabreichung von Placebos der klinische Interaktionsstil experimentell variiert.

Methodisches Vorgehen

Für die Studie sollen insgesamt 80 Personen untersucht werden, die unter Heuschnupfen Symptomen/Allergischer Rhinitis leiden. Es werden auch Personen eingeschlossen, die bereits anderweitige Medikation einnehmen, dies ist allerdings keine Voraussetzung. Die Medikation soll während der Placebo-Einnahme nicht verändert werden.

Als Grundparadigma für die Studie soll das Design der Studie von Schaefer und Kollegen [1] verwendet und für die vorliegende Studie angepasst werden. Die gemäß Selbstbericht an Heuschnupfen leidenden Teilnehmenden werden zu einem Erstgespräch eingeladen, in dem ihre Symptome erfragt werden und ihnen ein Placebo-Rational vermittelt wird. Dabei wird vor Eintreffen des Teilnehmenden bereits randomisiert, in welchem Interaktionsstil er/sie empfangen wird. Nach dem Gespräch wird der Teilnehmende zufällig einer Placebo-Bedingung zugeteilt. Teilnehmende in der aktiven Placebo-Bedingung müssen in den folgenden zwei Wochen zwei Mal täglich eine Placebo-Pille einnehmen. Nach Ablauf der zwei Wochen findet eine erneute Erhebung der Heuschnupfen Symptome aller Teilnehmenden statt.

Wissenschaftlicher Erkenntnisgewinn

Allergien und Heuschnupfen sind weltweit weit verbreitet und beeinträchtigen die Lebensqualität der Betroffenen [4, 5]. Hier stellen Placebos eine vielversprechende Behandlungsoption dar [12]. Gleichzeitig sind die zugrundeliegenden Mechanismen des Placeboeffekts weitestgehend ungeklärt [6]. Mit diesem Forschungsvorhaben könnte nicht nur die Befundlage zur Wirksamkeit von OLP weiter untermauert werden, sondern Implikationen für die Behandlung von Allergien abgeleitet und ein möglicher Mechanismus zur Verstärkung des Placebo-Effekts erklärt werden.

**D. Sind weitere Forscher an dem Forschungsvorhaben beteiligt?**	
Namen/Fakultät:	B.Sc. Verena Hofmann, Universität Koblenz-Landau, Fachbereich Psychologie, AG Klinische Psychologie und Psychotherapie<br>Prof. Dr. Julia Anna Glombiewski, Universität Koblenz-Landau, Fachbereich Psychologie, AG Klinische Psychologie und Psychotherapie<br>Prof. Dr. Ted Kaptchuk, Harvard Medical School, Program in Placebo Studies, Boston, USA<br>Prof. Dr. Irving Kirsch, Harvard Medical School, Program in Placebo Studies, Boston, USA

**E. Finanzierung?**		Ja	Nein
Forschungsträger:			**x**
Namen**:**			
Selbstfinanziert (im Rahmen einer Qualifikationsarbeit):		**x**	

**F. Wird vom Forschungsträger eine Ethik-Stellungnahme gefordert?**	Ja	Nein

**G. Dem Antrag liegen bei (bitte in einer PDF-Datei):** ***Bitte ankreuzen***	
Einwilligungserklärung (Pflicht)	x
Information und Aufklärung der Teilnehmer (Pflicht)	x
Erklärung, ob der Antrag bislang einer anderen Ethikkommission vorgelegt wurde. Falls ja, Stellungnahme beilegen	x
Unbedenklichkeitserklärung einer anderen Ethikkommission	
Alle Schritte des Forschungsablaufs in tabellarischer Form einschließlich Zusammenstellung der Art und Anzahl der Probanden, ihrer Rekrutierung, Instruktionen, Aufgaben, Fragebögen (nur Benennung), Instrumenten, die in der Studie verwendet werden	x
Einwilligungserklärung für Bild- und/oder Tonaufnahmen	
Debriefing bei Studien mit aktiver oder passiver Täuschung	

Anmerkung:
Details zur Einwilligungserklärung und zur Probandenaufklärung können den Ethik-Richtlinien der DGPs https://www.dgps.de/fileadmin/documents/Empfehlungen/berufsethische_richtlinien_dgps.pdf (3a-e; 6;9) sowie den DGPs-Richtlinien zur Antragstellung (Punkt C) entnommen werden: https://www.dgps.de/index.php?id=186

**H. Checkliste zur Studie**		
	Ja	Nein
**1. Freiwilligkeit:**<br>Ist die Freiwilligkeit der Teilnahme gewährleistet?	x	
**2. Geschäftsfähigkeit:**<br>Werden an der Studie Personen teilnehmen, die nicht selbst ihre Zustimmung zur Teilnahme geben können, da sie unmündig, eingeschränkt urteilsfähig oder urteilsunfähig sind (z.B. Babys, Kleinkinder, Personen unter 18 Jahren, Personen, die im juristischen Sinn nicht einwilligungsfähig sind)?		x

**3. Beeinträchtigte Personengruppe:**<br>Werden an der Studie Personen teilnehmen, die einer besonders verletzlichen Gruppe angehören (z.B. klinische Stichproben, Personen mit Lernschwäche, Personen im Klinik- oder Strafvollzugssetting, demente Personen, Personen in Altenheimen, Personen mit körperlichen Behinderungen)?	x	
**4. Abbruch:**<br>Wird den Teilnehmenden zugesichert, dass sie die Untersuchung jederzeit ohne Angabe von Gründen und ohne negative Konsequenzen abbrechen können?	x	
**5. Ein- und Ausschlusskriterien:**<br>Gibt es für die Probanden Ein- und/ oder Ausschlusskriterien?	x	
**6. Informierte Einwilligung:**<br>Wird schriftlich eine informierte Einwilligung eingeholt?	x	
**7. Aufklärung:**<br>Werden die Teilnehmenden vollständig über Ziele und Zwecke der Studie informiert/aufgeklärt?	x	
**8. Täuschung über Teilnahme:**<br>Ist es erforderlich, dass Personen an der Studie teilnehmen, ohne zu diesem Zeitpunkt über ihre Teilnahme informiert zu sein bzw. ohne ihre Einwilligung gegeben zu haben (z.B. bei experimentellen Felduntersuchungen, verdeckter Beobachtung) oder dass sie nicht hinreichend über Zweck und Inhalt der Studie informiert werden (die Offenlegung der Hypothesen zählt nicht dazu)?		x
**9. Aktive Täuschung über Inhalt, Zweck, Methode oder Setting:**<br>Werden Personen aktiv und gezielt über Inhalt, Zweck, Methode und/oder Setting der Studie getäuscht (z.B. indem falsche Zwecke vorgespiegelt, falsche Informationen gegeben, wichtige Informationen verschwiegen werden etc.)?		x
**10. Intimität/ Stigmatisierung:**<br>Werden Fragen zu Themen gestellt, die für die Befragten von intimer Natur sind (z.B. belastende persönliche Erlebnisse, Sexualität) oder deren Beantwortung als stigmatisierend wahrgenommen werden kann (z.B. zu illegalem oder deviantem Verhalten wie Drogenkonsum, Süchten oder Genussmittelmissbrauch aber auch zu politischen Überzeugungen)?		x
**11. Psychische Belastungen:**<br>Ist zu erwarten, dass die Teilnehmenden durch die Studie psychische Belastungen, Furcht, Erschöpfung oder andere negative Effekte erleiden?		x
**12. Körperliche Risiken:**<br>Werden mit den Teilnehmenden der Studie irgendwelche invasiven Messungen durchgeführt? Werden sie potenziell belastenden (z.B. Blut-, Speichelabgabe) oder potenziell schädlichen Prozeduren unterzogen? Werden ihnen körperliche Schmerzen zugefügt? Sind Nebenwirkungen zu erwarten?		x
**13. Substanzvergabe:**<br>Werden den Teilnehmenden in der Studie Medikamente, Placebos oder andere Substanzen verabreicht?	x	
**14. Vertraulichkeit:**<br>Werden personenbezogene Daten vertraulich behandelt und anonymisiert gespeichert?	x	
**15. Datenschutz:**<br>Ist die Datensicherheit der personenbezogenen Daten gewährleistet?	x	
**16. Datenschutzinformation:**<br>Werden die Teilnehmenden über die Datensicherheit der personenbezogenen Daten informiert?	x	
**17. Recht auf Datenlöschung:**<br>Bei Vorliegen einer Codeliste können die Teilnehmenden jederzeit die Löschung/Vernichtung Ihrer personen-bezogenen Daten verlangen und werden sie darüber informiert? Falls keine Codeliste vorliegt, kann direkt im Anschluss an die Untersuchung die Löschung verlangt werden?	x	
**18. Versicherungsschutz:**	x	

Besteht für die Teilnehmenden eine Wegeversicherung oder werden die Teilnehmenden darüber aufgeklärt, dass der Anfahrtsweg nicht versichert ist?		

Wenn Sie eine oder mehrere Fragen der Checkliste des Basisfragebogens im farbig hinterlegten Feld angekreuzt haben, reichen Sie bitte einen vollständigen Antrag mit den vollständigen Versuchsmaterialien ein. Gehen Sie im Vollantrag bitte insbesondere bei jeder Frage der Checkliste, bei der Sie im farbig hinterlegten Feld eine Ankreuzung vorgenommen haben darauf ein, warum dieser Aspekt der Studie notwendig ist und wie Sie dafür Sorge tragen werden, dass in Hinsicht auf diese Punkte die Ethikrichtlinien eingehalten werden. Zudem gehen Sie bitte auf den Kosten-Nutzen-Aspekt der Studie ein.

Anmerkung:
Genauere Informationen zu einzelnen Inhalten können der folgenden Internetseite entnommen werden: http://www.dgps.de/index.php?id=185

Ich bestätige, dass alle Angaben in diesem Antrag korrekt und gemäß den Richtlinien der Ethikkommission der DGPs erfolgt sind. Ich versichere außerdem, dass die vorliegenden Angaben nicht von den Angaben im Antrag an die betreffende Förderinstitution abweichen. Ich bin mir bewusst, dass die Verantwortung für die Einhaltung der Richtlinien letztendlich bei mir liegt.

Landau, 17.12.2019

Ort, Datum

Tobias Uhe

Unterschrift des hauptverantwortlichen Wissenschaftlers/Betreuer

Anlagen:

- Anhang A: Studieninformation
- Anhang B: Einverständniserklärung
- Anhang C: Studiendesign
- Anhang D: Eingesetzte Fragebögen
- Anhang E: Abschließende Information
- Anhang F: Begründung für ethisch evtl. problematische Entscheidungen
- Anhang G: Unbedenklichkeitsvota zu vorausgegangenen Untersuchungen
- Literaturverzeichnis

UNIVERSITÄT KOBLENZ · LANDAU

Studie zur Wirkung von Placebos bei Heuschnupfen

Studieninformationen

Studienleitung:

Dr. Tobias Kube
Universität Koblenz-Landau
AG Klinische Psychologie und Psychotherapie
Ostbahnstr. 10, 76829 Landau
E-Mail: kube@uni-landau.de

Sehr geehrte Studieninteressentin, sehr geehrter Studieninteressent,

in unserer Arbeitsgruppe wird derzeit eine Studie zur Wirkung von Placebos bei Heuschnupfen statt. Wir möchten Sie im Folgenden darüber informieren und Sie um Ihre Teilnahme an der Studie bitten.

Worum geht es in der Studie?
Unter „Placebo-Effekten“ fasst man positive Effekte von Behandlungen zusammen, die keine medizinisch-pharmakologisch wirksamen Inhaltsstoffe zusammen enthalten. Lange Zeit ging man davon aus, dass Placebo-Effekte nur dann auftreten können, wenn Patientinnen und Patienten (fälschlicherweise) glauben, ein „echtes“ Medikament zu erhalten. Neuere Forschung hat diese Annahme in Frage gestellt: Inzwischen ist bekannt, dass auch offen verabreichte Placebos positive Wirkungen erzielen können. In unserer Studie wollen wir die Wirkung von offen verabreichten Placebos bei allergischer Rhinitis (allgemein bekannt als „Heuschnupfen“) untersuchen und verstehen, wie ihre Wirkung zustande kommt.

Was bedeutet die Teilnahme an der Studie für mich?
Wenn Sie an der Studie teilnehmen, werden Sie per Zufall entweder der Placebo-Gruppe zugewiesen oder der Kontrollgruppe. Wenn Sie der Placebo-Gruppe zugewiesen werden sollten, erhalten Sie von uns Placebo-Tabletten, die Sie zwei Wochen lang zwei Mal täglich einnehmen. Nach Ablauf der zwei Wochen findet ein erneuter Termin hier in der Ostbahnstr. statt, bei dem Sie über Ihre Erfahrungen mit dem Placebo befragt werden und in einem Fragebogen angeben, wie sich Ihre Symptome entwickelt haben. Falls Sie der Kontrollgruppe zugewiesen werden sollten, erhalten Sie zunächst zwei Wochen lang keine Behandlung von uns. Nach zwei Wochen findet dann, wie in der Placebo-Gruppe, ein erneuter Termin statt, bei dem Sie bzgl. der Entwicklung Ihrer Symptome befragt werden. Wenn Sie möchten, erhalten Sie im Anschluss an dieses Gespräch dann ebenfalls die Placebo-Tabletten, die Sie zwei Wochen lang zwei Mal täglich einnehmen können. Nach diesen Wochen würde dann erneut ein Termin stattfinden, bei dem Sie über Ihre Erfahrungen mit den Placebo-Tabletten befragt würden. Jeder Termin bei uns würde ca. 30 Minuten in Anspruch nehmen. Sollten wir im Rahmen unserer Befragung klinisch auffällige Befunde feststellen, können wir Sie darüber gern informieren, wenn Sie dies wünschen. Sollten Sie eine solche Rückmeldung wünschen, sprechen Sie bitte die Studienleitung an.

Warum sollte ich an der Studie teilnehmen?
Mit Ihrer Teilnahme an der Studie helfen Sie dabei, besser zu verstehen, wie offen verabreichte Placebos wirken. Basierend auf vorangegangenen Studien zu offen verabreichten Placebos bei Heuschnupfen besteht die realistische Möglichkeit, dass die Placebo-Behandlung sich positiv auf

Ihre Symptomentwicklung auswirkt. Mit Ihrer Teilnahme helfen Sie außerdem dabei, besser zu verstehen, wie sich die Behandlung von Heuschnupfen verbessern lässt.

Mögliche Nachteile und Risiken
Ein großer Vorteil von Placebos ist, dass Sie keine Nebenwirkungen hervorrufen. Außer dem Zeitaufwand sind daher keine Nachteile durch die Studienteilnahme zu erwarten.

Datenschutz und Freiwilligkeit
Die Teilnahme an der Studie ist freiwillig. Der Rücktritt von Ihrem Einverständnis zur Teilnahme ist jederzeit ohne Angabe von Gründen möglich und mit keinerlei Nachteilen verbunden. Die erfassten Daten werden pseudonymisiert und streng vertraulich entsprechend den Regeln des Datenschutzes (Bundesdatenschutzgesetz §40) und der Schweigepflicht behandelt. Das bedeutet, dass Ihnen auf einer Liste ein Code zugeordnet wird, unter dem die Daten elektronisch abgespeichert werden. Diese Zuordnungsliste wird unter Verschluss gehalten. Nach Abschluss der Datenerhebung (spätestens zum 30.06.2021) wird der Zuordnungsschlüssel vernichtet, Ihre Daten liegen dann in anonymisierter Form vor. Dies bedeutet, dass ein Rückschluss von den Daten auf Ihre Person nicht mehr möglich ist. Die anonymisierten Daten werden im Rahmen gesetzlicher Bestimmungen noch 10 Jahre aufbewahrt. Solange der Zuordnungsschlüssel existiert, d.h. bis zum Abschluss der Datenerhebung, können Sie jederzeit die Löschung (oder Einschränkung der Verarbeitung) Ihrer erhobenen Daten verlangen. Danach kann Ihr Name den Daten nicht mehr zugeordnet werden, sodass eine Löschung nicht mehr möglich ist. Ihre Daten werden ausschließlich für Auswertungen im Rahmen der Studie verwendet. Die anonymisierten Ergebnisse der Studie können als wissenschaftliche Publikationen veröffentlicht werden. Ihre Einverständniserklärung wird getrennt von den im Rahmen der Studie erhobenen Daten aufbewahrt und nach Abschluss der Studie (spätestens zum 30.06.2021) vernichtet.

Sollten Sie noch weitere Fragen zu unserer Studie haben, zögern Sie nicht, uns zu kontaktieren. Die Kontaktdaten der Studienleitung sind:

Dr. Tobias Kube

Universität Koblenz-Landau

AG Klinische Psychologie und Psychotherapie
Ostbahnstr. 10, 76829 Landau
E-Mail: kube@uni-landau.de
Telefon: 06341-28035652

Studie zur Wirkung von Placebos bei Heuschnupfen

Einverständniserklärung

Studienleitung:

Dr. Tobias Kube
Universität Koblenz-Landau
AG Klinische Psychologie und Psychotherapie
Ostbahnstr. 10, 76829 Landau
E-Mail: kube@uni-landau.de

Ich bin über Wesen, Bedeutung und Tragweite der o.g. Studie unter der Leitung von Dr. Tobias Kube aufgeklärt worden. Ich hatte ausreichend Gelegenheit, mich bei der Versuchsleitung über die Studie zu informieren, sowie auftretende Fragen zu stellen. Diese wurden mir von der Versuchsleitung verständlich beantwortet. Mir ist bekannt, dass ich mich bei weiteren Fragen an die Studienleitung unter den oben genannten Kontaktdaten wenden kann.

Mir ist bekannt, dass diese Studie in erster Linie der Wissenserweiterung dient und gegebenenfalls auch keinen persönlichen Vorteil für mich bringen kann. Ich weiß, dass ich die Studienleitung ansprechen kann, falls ich den Wunsch habe, über evtl. klinisch auffällige Befunde informiert zu werden.

Mit meiner Unterschrift erkläre ich, dass ich das Vorhaben und die Information verstanden habe und freiwillig an der Studie teilnehme. Ich habe verstanden, dass ich jederzeit ohne Angabe von Gründen aus der Studie ausscheiden kann, ohne dass mir persönliche Nachteile entstehen. Auch der Versuchsleiter oder die Versuchsleiterin kann die Studie jederzeit beenden.

Mir ist bekannt, dass meine Daten pseudonymisiert und streng vertraulich entsprechend den Regeln des Datenschutzes (Bundesdatenschutzgesetz §40) und der Schweigepflicht behandelt werden. Das bedeutet, dass meiner Person auf einer Liste ein Code zugeordnet wird, unter dem die Daten elektronisch abgespeichert werden. Diese Zuordnungsliste wird unter Verschluss gehalten. Nach Abschluss der Datenerhebung (spätestens zum 30.06.2021) wird der Zuordnungsschlüssel vernichtet, meine Daten liegen dann in anonymisierter Form vor. Dies bedeutet, dass ein Rückschluss von den Daten auf meine Person nicht mehr möglich ist. Die anonymisierten Daten werden im Rahmen gesetzlicher Bestimmungen noch 10 Jahre aufbewahrt. Solange der Zuordnungsschlüssel existiert, d.h. bis zum Abschluss der Datenerhebung, kann ich jederzeit die Löschung (oder Einschränkung der Verarbeitung) der individuellen erhobenen Daten verlangen. Danach kann mein Name den Daten nicht mehr zugeordnet werden, sodass eine Löschung nicht mehr möglich ist. Meine Daten werden ausschließlich für Auswertungen im Rahmen der Studie verwendet. Die anonymisierten Ergebnisse der Studie können als wissenschaftliche Publikationen veröffentlicht werden.

Ich habe das Recht,

- Auskunft (gemäß Art. 15 DS-GVO) zu erhalten, einschließlich unentgeltlicher Überlassung einer Kopie,
- ggf. deren Berichtigung (gemäß Art. 16 DS-GVO) zu verlangen,
- ggf. deren Löschung zu verlangen, solange dem keine Aufbewahrungspflichten (gemäß Art. 17 DS-GVO) entgegenstehen,
- die Einschränkung der Verarbeitung zu verlangen (gemäß Art. 18 DS-GVO),
- die Daten einem anderen ohne Behinderung zu übermitteln (Datenübertragung gemäß Art. 20 DS-GVO),
- Einwendung gegen die Nutzung für wissenschaftliche Zwecke (über die direkten Zwecke der Studie hinaus) zu erheben.

Die Kontaktdaten der Studienleitung sind:
Dr. Tobias Kube, Universität Koblenz-Landau, AG Klinische Psychologie und Psychotherapie, Ostbahnstr. 10, 76829 Landau, E-Mail: kube@uni-landau.de, Telefon: 06421-2823341

Die Kontaktdaten der zuständigen Datenschutz-Aufsichtsbehörde Rheinland-Pfalz lauten:
Der Landesbeauftragte für den Datenschutz Rheinland-Pfalz, Hintere Bleiche 34, 55116 Mainz
Postanschrift:
Postfach 30 40
55020 Mainz

Die Kontaktdaten des zuständigen Datenschutzbeauftragten vom Campus Landau lauten:
Universität Koblenz-Landau, Datenschutzbeauftragte Dr. Susanne Weis, Campus Landau, Fortstr. 7, 76829 Landau, datenschutz@uni-landau.de

Hiermit erkläre ich, dass ich über Ziele und Ablauf der Studie informiert wurde und diese verstanden habe. Ich erkläre hiermit freiwillig meine Studienteilnahme.

Name, Vorname: ______________________________

______________________________ Ort, Datum und Unterschrift der Studienleitung

______________________________ Ort, Datum und Unterschrift des Studienteilnehmers

Anhang C: Studiendesign und Ablauf

Design

Es handelt sich um ein 2x2 Design, in dem die Behandlung (Placebo vs. No Treatment) sowie der klinische Interaktionsstil (angereichert vs. limitiert) variiert werden.

Prätestung

Zu Beginn werden die Symptome der Teilnehmenden und deren Schwere mithilfe des von Schaefer und Kollegen [7] entwickelten Fragebogens erhoben. Im Anschluss wird den Teilnehmenden ein Placebo-Rational sowie das weitere Vorgehen vermittelt. Das Verständnis insbesondere der Qualität der Placebo-Pillen wird im Nachhinein abgefragt, um sicherzustellen, dass die Teilnehmenden wissen, dass sie keine medizinisch-pharmakologisch wirksame Substanz einnehmen werden. Darauf folgt die zufällige Zuteilung zu einer der beiden Behandlungs-Bedingungen: Placebo vs. No Treatment (NT) Kontrollgruppe und ggf. die Übergabe der Placebo-Pillen für die kommenden zwei Wochen. Sollten die Teilnehmenden der Placebo-Gruppe zugewiesen werden, werden folgende Informationen zu Placebos und Placebo-Effekten vermittelt:

- In zwei vorherigen Studien (Schäfer et al., 2016, 2018) hat sich ein offen verabreichtes Placebo als wirksame Behandlungsmöglichkeit bei Heuschnupfen erwiesen, das die Symptomatik positiv beeinflussen konnte.
- Als mögliche Wirkfaktoren dieses Effekts werden aktuell verschiedene Aspekte in Betracht gezogen, u.a. Lernmechanismen wie klassische Konditionierung.
- Es kann zwar hilfreich sein, positive Erwartungen an die Einnahme von Placebos zu haben, es aber ist auch völlig normal ist, skeptisch gegenüber Placebos zu sein.
- Im Hinblick auf Adhärenz wird den Teilnehmenden mitgeteilt, dass es sehr wichtig ist, dass sie das Placebo während des Studienzeitraums zwei Mal täglich einnehmen, da vorherige Forschung gezeigt hat, dass sich Placebos v.a. dann günstig auf den Symptomverlauf auswirken, wenn sie regelmäßig eingenommen werden.
- Zuletzt werden die Teilnehmenden darauf hingewiesen, dass Menschen unterschiedlich stark und schnell auf Placebos ansprechen können und dass es daher sein kann, dass sie nicht direkt nach der ersten Einnahme schon einen Effekt merken werden.

Experimentelle Bedingungen

Die Variation des klinischen Interaktionsstils erfolgt anhand nachfolgend aufgelisteter Merkmale. Wichtig ist den Antragstellenden dabei die Feststellung, dass die limitierte Bedingung nicht als bewusstes Negativbeispiel bzgl. der klinischen Interaktion geplant wird. D.h. es soll nicht künstlich versucht werden, eine besonders ungünstige klinische Begegnung zu erzeugen. Vielmehr ist geplant, dass die limitierte Bedingung ein neutrales, sachliches Gespräch darstellt, während in der angereicherten Bedingung zusätzliche positive Merkmale eingebaut werden und die klinische Interaktion als besonders herzlich und personalisiert geplant wird.

Faktor	Limitiert	Angereichert
**Verbale Kommunikation**	• Kurze Begrüßung ohne namentliche Vorstellung<br>• Wenig Rückmeldung/-fragen<br>• Wenig Fragen stellen<br>• Gespräch hat eher standardisierten Interviewcharakter mit Fokus auf Prozedur<br>• Nüchterne, sachliche Sprache	• Persönliche Begrüßung mit namentlicher Vorstellung<br>• Raum für Rückfragen und Rückmeldungen; Fragen stellen<br>• Fachbegriffe vermeiden bzw. erläutern<br>• Patientengerechte, empathische und freundliche Sprache<br>• Fragen zur Symptomatik sowie zur Lebensqualität<br>• Bisherige Versuche zur Symptomreduktion loben<br>• Dankbarkeit für Teilnahme
**Non-verbale Kommunikation**	• Wenig Blickkontakt, nebenbei Bogen ausfüllen<br>• Wenig Gestik, wenig Mimik (neutraler Gesichtsausdruck)<br>• Pflicht- und sachorientierter Eindruck; Grundatmosphäre eher nüchtern	• Viel Blickkontakt<br>• Aufrechte, zugewandte Körperhaltung<br>• Bestätigende, validierende Gestik und Mimik, freundliches Lächeln<br>• Freundliche Begrüßung mit Hand geben<br>• Personenorientiertes Setting; Grundatmosphäre warm und fürsorglich
**Umgebungs- oder Kontextfaktoren**	• Sitzposition hinter Schreibtisch, Pat. davor sitzend<br>• Kein Namensschild<br>• Unpersönlicher Kittel<br>• Keine persönlichen Gegenstände im Raum, recht steriler Behandlungsraum	• Sitzposition an gemeinsamem Besprechungstisch<br>• Namensschild<br>• Glas Wasser auf dem Tisch, evtl. mit Karaffe<br>• Blumen auf Tisch<br>• Im Hintergrund Bücherregal mit Fachliteratur<br>• Private Bilder im Hintergrund im Bücherregal<br>• Kittel, aber persönlicher Kleidungsstil erkennbar

Entsprechend der Variation der klinischen Interaktion gibt es zwei NT Kontrollgruppen (jeweils $n = 20$). Beide Kontrollgruppen nehmen keine Placebos ein. Die erste NT Kontrollgruppe erhält ein angereichertes Erstgespräch zur Erhebung der Symptome, Symptomschwere und Beeinträchtigung durch die Symptome im Alltag sowie ein Abschlussgespräch zwei Wochen später. Die zweite NT Kontrollgruppe durchläuft ein limitiertes Erstgespräch mit demselben Inhalt sowie ein Abschlussgespräch nach ebenfalls zwei Wochen.

Dem gegenüber stehen die beiden Gruppen, die ein Placebo erhalten (jeweils $n = 20$). Beide aktiven Experimentalgruppen nehmen nach dem Erstgespräch über die Dauer von zwei Wochen zwei Mal täglich jeweils eine Placebo-Pille ein. Die erste Experimentalgruppe erhält ein angereichertes Erstgespräch sowie ein Abschlussgespräch nach Beenden der Placebo-Einnahme mit denselben Inhalten wie die Gespräche der Kontrollgruppen. Die zweite Experimentalgruppe durchläuft ein limitiertes Erstgespräch sowie ein Abschlussgespräch nach Beenden der Placebo-Einnahme mit demselben Inhalt wie die Gespräche der Kontrollgruppen.

<u>Posttestung</u>

Nach Ablauf der zwei Wochen, in denen die Teilnehmenden der aktiven Placebo-Bedingung zwei Mal täglich je eine Placebo-Pille eingenommen haben und die Teilnehmenden der NT Kontrollgruppe keine Placebo-Behandlung erhalten haben, werden die Symptome erneut anhand des Fragebogens von Schaefer und Kollegen [7] erhoben. Außerdem findet ein Manipulations-Check in dem Sinne statt, dass die Teilnehmenden die Interaktion mit der behandelnden Person ebenfalls in einem Fragebogen bewerten sollen. Übrige Placebo-Pillen werden der behandelnden Person zurückgegeben.

Anhang D: Eingesetzte Fragebögen

- Fragen zur Heuschnupfen-Symptomatik [5, 7]
- Adaptierte Version des Pain Disability Index (PDI) zur Einschätzung der Beeinträchtigung durch Heuschnupfen-Symptome im Alltag
- PHQ-9 als Screening Instrument für depressive Symptome
- Fragen zur Wahrnehmung des klinischen Interaktionsstils
- Fragen zu den Behandlungserwartungen
- Fragen zum Wissen über Placebos
- Soziodemographische Fragen zu Alter, Geschlecht, Bildungsstand, beruflicher Tätigkeit, Familienstand

UNIVERSITÄT KOBLENZ · LANDAU

Studie zur Wirkung von Placebos bei Heuschnupfen

Abschließende Information

Studienleitung:
Dr. Tobias Kube
Universität Koblenz-Landau
AG Klinische Psychologie und Psychotherapie
Ostbahnstr. 10, 76829 Landau
E-Mail: kube@uni-landau.de

Sehr geehrte Studienteilnehmerin, sehr geehrter Studienteilnehmer,

herzlichen Dank dafür, dass Sie an unserer Untersuchung teilgenommen haben. Wir möchten Ihnen zum Abschluss noch einige weitere Hinweise zur Studie geben.

In unserer Studie möchten wir suchen, inwiefern die Wirkung von offen verabreichten Placebos beeinflusst wird durch die Interaktion zwischen Patient/in und Behandler/in. Daher haben wir in unserer Studie zwei Formen der klinischen Interaktion getestet: eine sachliche, wenig personalisierte Interaktion oder eine angereicherte, besonders herzliche und personalisierte Interaktion. Es wurde per Zufall entschieden, ob Sie den einen oder anderen Interaktionsstil im Gespräch mit der Behandlerin erhalten.

Wenn Sie noch weitere Fragen zu der Untersuchung haben zögern Sie nicht, uns anzusprechen.

Anhang F: Begründungen für ethisch evtl. problematische Entscheidungen

Beeinträchtigte Stichprobe sowie Ein- und Ausschlusskriterien

An der Studie sollen nur Personen teilnehmen, die durch Heuschnupfen Symptome gemäß Selbstbericht belastet sind. In vorherigen Studien [1] wurde gezeigt, dass OLP bei dieser Stichprobe wirksam sein können. Die Teilnahme der beeinträchtigten Personen kann dazu verhelfen, Interventionen abzuleiten, die perspektivisch die Behandlungsmöglichkeiten für diese Gruppe optimieren können. Dies ist besonders vor dem Hintergrund mangelnder präventiver Maßnahmen und einem gleichzeitigen Anstieg der Prävalenzraten allergischer Erkrankungen zu betrachten [10].

Aus den obigen Darstellungen ergibt sich die Notwendigkeit von Einschlusskriterien, um diese Patientenstichprobe untersuchen zu können.

Die Einschlusskriterien lauten:

- Vorliegen von Heuschnupfen Symptomen im Selbstbericht
- mindestens 18 Jahre alt
- ausreichend gute Deutschkenntnisse

Ausreichend gute Deutschkenntnisse sind notwendig, da ein Großteil der experimentellen Manipulation sprachbasiert ist und ein ausreichendes Verständnis hierfür sichergestellt werden muss.

Substanzvergabe

Wesentlicher Bestandteil dieser Studie ist die Verabreichung von Placebo-Tabletten an die eine Hälfte der Teilnehmenden, während die andere Hälfte diese nicht erhält. Dies ist notwendig, die Haupteffekte der Substanz und der klinischen Interaktion sowie deren Interaktion zu untersuchen. Die Teilnehmenden werden im Vorfeld darüber aufgeklärt, dass es sich bei den Placebos ohne pharmakologisch unwirksame Tabletten handelt, sodass sie ihr Einverständnis unter vollumfänglicher Aufklärung geben können. Die Zusammensetzung der Placebo-Tabletten stellt kein gesundheitliches Risiko dar.

Anhang G: Unbedenklichkeitsvota zu vorausgegangenen Untersuchungen

In den letzten Jahren hat unsere Arbeitsgruppe an der Philipps-Universität Marburg diverse Studien zu Placebo- und Noceboeffekten durchgeführt. Diese wurden von der lokalen Ethikkommission als ethisch unbedenklich eingeschätzt. Die zwei jüngsten Studien, in denen Wirkfaktoren von Open-Label Placebos untersucht wurden, waren die folgenden:

- Projektnamen: „Hoffnung und Erwartungen als Wirkfaktoren von Open-Label Placebos - Eine experimentelle Studie zur Schmerzwahrnehmung“; „Wirkfaktoren bei offenen und verdeckten Placebos - Eine experimentelle Studie zur Induktion von Traurigkeit“
- Beteiligte Ethikkommissionen: Ethikkommission des Fachbereichs Psychologie der Philipps-Universität Marburg

Literaturverzeichnis

1. Kaptchuk, T. J. (2018). Open-label Placebo: Reflections on a research agenda. Perspectives in Biology and Medicine, 61, 311-334. doi: 10.1353/pbm.2018.0045

2. Locher, C., Nascimento, A. F., Kirsch, I., Kossowsky, J., Meyer, A., & Gaab, J. (2017). Is the rationale more important than deception? A randomized controlled trial of open-label placebo analgesia. PAIN, 0, 1-9. doi: 10.1097/j.pain. 0000000000001012

3. Kaptchuk, T. J., Friedlander, E., Kelley, J. M., Sanchez, M. N., Kokkotou, E., Singer, J. P., … & Lembo, A. J. (2010). Placebos without deception: A randomized controlled trial in irritable bowel syndrome. PLoS ONE, 5, 1-7. doi: 10.1371/journal.pone.0015591

4. Carvalho, C., Caetano, J. M., Cunha, L., Rebouta, P., Kaptchuk, T. J., & Kirsch, I. (2016). Open-label-placebo treatment in chronic low back pain: A randomized controlled trial. Pain, 157, 2766-2772. doi: 10.1097/j.pain. 0000000000000700

5. Schaefer, M., Harke, R., & Denke, C. (2016). Open-label placebos improve symptoms in allergic rhinitis: A randomized controlled trial. Psychotherapy and Psychosomatics, 85, 373-374. doi: 10.1159/000447242

6. Kaptchuk, T. J., & Miller, F. G. (2018). Open label placebo: Can honestly prescribed placebos evoke meaningful therapeutic benefits? British Medical Journal, 363, 1-3. doi: 10.1136/bmj.k3889

7. Schaefer, M., Sahin, T., & Berstecher, B. (2018). Why do open-label placebos work? A randomized controlled trial of an open-label placebo induction with and without extended information about the placebo effect in allergic rhinitis. PLoS ONE, 13(3), 1-14. doi: 10.1371/journal.pone.0192758

8. Enck, P., Bingel, U., Schedlowski, M., & Rief, W. (2013). The placebo response in medicine: Minimize, maximize or personalize? Nature Reviews Drug Discovery, 12, 191-204. doi: 10.1038/nrd3923

9. Kaptchuk, T. J., Kelley, J. M., Conboy, L. A., Davis, R. B., Kerr, C. E., Jacobson, E. E., … & Lembo, A. J. (2008). Components of placebo effect: Randomised controlled trial in patients with irritable bowel syndrome. British Medical Journal, 336, 999-1003. doi: 10.1136/bmj.39524.439618.25

10. Bergmann, K.-C., Heinrich, J., & Niemann, H. (2015). Current status of allergy prevalence in Germany. Allergo Journal International, 25, 6-10. doi: 10.1007/s40629-016-0089-1

11. Seidman, M.D., Gurgel, R.K., Lin, S.Y., Schwartz, S.R, Baroody, F.M., Bonner, J.R., … & Nnacheta, L.C. (2015). Clinical practice guideline: Allergic rhinitis. Otolaryngology – Head and Neck Surgery, 152, S1-S43. doi: 10.1177/0194599814561600

12. Wedi, B. (2019). Der Placeboeffekt in der Allergologie: Die ideale Therapieoption? Allergolgoie, 42, 155-156. doi: 10.5414/ALX02084e

13. Enck, P., Bingel, U., Schedlowski, M., & Rief, W. (2013). The placebo response in medicine: Minimize, maximize or personalize? Nature Reviews Drug Discovery, 12, 191-204. doi: 10.1038/nrd3923

14. Hoenemeyer, T. W., Kaptchuk, T. J., Mehta, T. S., & Fontaine, K. R. (2018). Open-label placebo treatment for cancer-related fatigue: A randomized-controlled clinical trial. Scientific Reports, 8, 2784-2791. doi: 1038/s41598-018-20993-y
