# Supplementary material for: Providing open-label placebos remotely—A randomized controlled trial in allergic rhinitis
Source: PLoS One. 2021 Mar 11;16(3):e0248367. doi: 10.1371/journal.pone.0248367 (PMC7951912; doi:10.1371/journal.pone.0248367)
Supplement: Attachment — Submitted filename: Response to Reviewers 012321.docx [file pone.0248367.s005.docx]

**Response to Reviewers**

The authors would like to thank the reviewers for their valuable and helpful feedback. Based on the referees’ comments, we revised the manuscript and feel it has improved as a result.

For your convenience, your original comments are repeated here in bold font, with our replies in standard font. The revised or new passages in the manuscript are quoted here in Italics; for revising the manuscript, we used the track-changes mode, so you can follow all changes made to the manuscript. In addition, we also uploaded a “clean” version of the manuscript without marked changes.

**Reviewer #1**

**The authors investigated the placebo effect in an allergic rhinitis randomized controlled trial by providing placebo remotely and virtual clinical encounter. They concluded it was possible to provide open-label placebo and enhance the encounter remotely, but the effectiveness might be lower than physical patient-provider interaction. In general, this is a well-designed study, the authors described their findings with details. The study is of novelty, especially in the COVID-19 pandemic period.**

Thank you very much for appreciating the value of our study!

**My major concern is the small sample size of this study, as only 54 patients were enrolled in this 4-arm clinical trial and an average of 14 cases in each arm. I’m not sure if it is qualified to discriminate the potential differences among the 4 subgroups, although the authors have acknowledged it as a limitation in the discussion.**

We agree that the small sample size is a significant limitation of the present study. To take this better into account, we elaborated on this issue in more detail in the limitations section. Specifically, we revised the beginning of the limitations section in response to your comment as follows (p. 32):

*The most significant limitation is the small sample size (N = 54) with less than 30 participants per treatment group and less than 15 persons in each of the four arms, thus limiting the chance to uncover potential differences between the groups. Yet, it should be noted that previous OLP studies examining people with allergic rhinitis had even smaller sample sizes (i.e. N = 25 in [16] and N = 46 in [17]), and based on the large effect sizes revealed in that prior work, the a-priori power analysis indicated a minimum sample size of 52 participants, which was reached, actually. Nevertheless, we acknowledge that the power analysis was based on the original plan to provide OLPs in a regular clinical encounter with physical contact, and under the assumption that OLPs are less effective when provided remotely, the current sample size might not have been large enough to detect significant effects of the placebo over TAU.*

**Meanwhile, the manuscript can be shortened and avoid narrating repeatedly.**

In response to your comment, we carefully went over the entire manuscript again and aimed to shorten it wherever possible without losing necessary information. Accordingly, we shortened the manuscript at several points, i.e., the description of additional results in the control group (p. 26), the discussion of the results (p. 29 and p. 31), and the implications for future work (p. 32). We feel these shortenings improved the overall flow and conciseness of the text, thanks to your comment.

**Reviewer #2**

**The authors presented the interaction between OLP (with and without TAU) and encounter (augmented and limited) during COVID-19 pandemic, and found that the remote provision of OLP is feasible, but their effectiveness might be lower than those found in previous research for OLP provided within a physical encounter. There are several questions,**

**1. Augmented and limited encounters may have some impact on patients’ psychology, do you think there are some impact on the effects, especially in control & limited groups?**

Thank you for that interesting question. We are not entirely sure, unfortunately, what you mean by the “patients’ psychology”. What we can say for sure, though, is that the type of the encounter (augmented vs. limited) had no influence on several psychological variables. In particular, we tested whether the two encounter styles differed in their treatment expectancies and the extent to which they felt informed about placebos. In doing so, we found no differences between the two encounter styles. The respective section of the manuscript reads (pp. 20):

*A 2x2 (Treatment by Encounter) ANOVA indicated that the two treatment groups did not differ in their treatment expectancies, F(1, 50) = 0.225; p = .637; ɳ²p = .004, and the extent to which they felt informed about placebos, F(1, 50) < 0.001; p = .991; ɳ²p < .001. Similarly, people from the augmented vs. limited encounter did not differ in their expectancies F(1, 50) = 0.035; p = .853; ɳ²p = .001, and the degree to which they felt informed about placebos, F(1, 50) = 0.965; p = .331; ɳ²p = .019. Also, there was no Treatment by Encounter interaction for expectancies (F(1, 50) = 1.338; p = .253; ɳ²p = .026) and feeling informed about placebos (F(1, 50) = 0.965; p = .331; ɳ²p = .019). As these measures were rated prior to randomization, no group differences were to be expected here.*

Except for these variables and the perception of the clinical encounter, we did not assess further psychological variables, making it difficult to answer your question beyond the above. We agree, though, that it is possible that some unmeasured psychological variables, such as negative affect, could have influenced the results. Accordingly, thanks to your comment, we added the following sentence to the limitations section (p. 33) to point to that issue:

*Moreover, it would have been interesting to assess the effects of the augmented vs. limited encounter on additional psychological variables, such as negative affect [36], to get a broader understanding of the effects of the encounter and to see whether such psychological variables could have influenced the effects of the placebo.*

**2. Previous studies have showed that network may help patients to use medicine regularly, and benefit to symptoms control. However, in this study, the authors have different results. I want to know that in no treatment subgroup, did the patients have the TAU or OLP regularly? If they took the medicine (OLP or TAU) as other subgroups, changes of symptom score may origin from the higher score in no treatment group and the lower score in regular treatment group.**

In both the OLP+TAU and the TAU alone group, patients were allowed to continue to take their regular medication if there was any. Some of the patients took this medication regularly, others took it on demand. Importantly, this was pertinent in both treatment groups (OLP+TAU and the TAU alone) and the groups did not differ in their frequency patterns regarding medication intake. Nevertheless, you are perfectly right that medication intake did influence the results, in the sense that it differentially affected the effectiveness of OLP vs. TAU, as presented on p. 24 in the results section. In particular, in people from the OLP+TAU group, symptom reduction was greatest if they did not take any regular medication for their allergic symptoms, whereas in the TAU group, symptom improvement was greatest if they took medication on demand.

**3.What’s the frequency of regular treatment, treatment on demand and no treatment in four subgroups of OLP-augmented, OLP-limited, TAU-augmented, and TAU-limited? Do you think there are some impact on the results of the four subgroups respectively?**

The results of the frequency of regular medication for the four groups seperately are presented in Table 2, as highlighted below. As can be seen there, most participants, across groups, took medication on demand. Since the distribution of the frequencies was not significant different across groups (*χ*²= 2.258; *p*=.894), it is unlikely that it influenced the results beyond what we discussed in response to your second question above.

Table 2

*Sample Characteristics*

| **Variable** | **OLP-augmented (n=14)** | **OLP-limited (n=14)** | **TAU-augmented (n=13)** | **TAU-limited (n=13)** | **Group differences** |
| --- | --- | --- | --- | --- | --- |
| Mean age in years (SD) | 26.8 (10.1) | 27.1 (11.0) | 32.2 (11.4) | 39.8 (17.5) | *F*(3, 50)=3.014; *p*=.039; *η²*=.153 |
| % female | 64.3 | 64.3 | 69.2 | 76.9 | *χ*²=0.661; *p*=.882 |
| Educational degree, %  Primary education  High school  University degree | 0  57.1  42.9 | 0  71.4  28.6 | 7.7  30.8  61.5 | 15.4  61.5  33.1 | *χ*²= 9.595; *p*=.384 |
| Employment status, %  University student  Employed  Self-employed  Other | 78.6  14.3  7.1  0 | 64.3  28.6  0  7.1 | 53.8  46.2  0  0 | 46.2  15.4  23.1  15.4 | *χ*²= 16.379; *p*=.174 |
| Current allergic medication, %  Regular intake  On demand  No medication | 42.9  42.9  14.2 | 28.6  42.9  28.6 | 30.8  38.5  30.8 | 30.8  53.8  15.4 | *χ*²= 2.258; *p*=.894 |
| Allergic symptoms at baseline, M (SD)^1^ | 15.1 (4.8) | 16.2 (2.9) | 15.6 (4.7) | 15.9 (3.4) | *F*(3, 50)=0.181; *p*=.909; *η²*=.011 |
| Impairment at baseline, M (SD)^2^ | 25.5 (14.0) | 25.5 (11.7) | 25.0 (12.4) | 27.6 (12.2) | *F*(3, 50)=0.112; *p*=.953; *η²*=.007 |

*Note*: OLP = open-label placebo; TAU = treatment as usual

^1^ Total score ranges from 0 to 18

^2^ Total score ranges from 0 to 70

**Reviewer #3**

**A two-arm placebo controlled randomized clinical trial was conducted to compare TAU alone to OLP+TAU and clinical encounter (augmented vs. limited). Primarily ANOVA models were used to test for differences in outcomes between treatments and clinical encounters. Participants from all treatment groups showed significant symptom reduction from baseline to two weeks later, but OLP had no incremental effect over TAU.

Minor revision:
1- Indicate the statistical testing method used in G*Power to justify the sample size.**

In response to your comment, we added the statistical testing method to the description of the power analysis. Accordingly, we revised the respective section as follows (p. 7):

*Accordingly, we expected a large effect (f = 0.4) of OLP (vs. TAU) on symptom improvement, and the power analysis using G*Power for an analysis of variance with fixed effects, special, main effects, and interactions indicated a required sample size of at least 52 people (alpha: .05; power: .80).*
 **2- Indicate if the following procedure was followed when testing main and interaction effects. If the interaction effect is significant, provide an interpretation of the results, but do not test main effects because the tests for main effects are uninteresting in light of significant interactions. If interaction effects are non-significant, drop the interaction effects from the model and test the main effects. Determining which results to present when testing interactions is often a multi-step process.**

Thank you for that comment. In response to your comment, we added the following sentences to the description of the statistical procedure (p. 18):

*When testing main and interaction effects, the following procedure was followed: If the interaction effect was significant, we provided an interpretation of the results, but did not test main effects because the tests for main effects are uninteresting in light of significant interactions. If interaction effects were non-significant, we dropped the interaction effects from the model and tested the main effects.*

In addition, we revised the presentation of the results from the ANOVAs to take your comment into account. This refers to the presentation of the main analysis for the primary endpoint on p. 22, the additional analyses (controlling for baseline symptoms and medicaiton use) on pp. 23 and 24 as well as the the results for the secondary endpoint (p. 25) and the switch-over in the control group (p. 26).
